# Cumulative risk of revision after primary total hip arthroplasty in registries internationally: systematic review and meta-analysis of selected hip stems and cups

**DOI:** 10.1530/EOR-2024-0020

**Published:** 2025-05-05

**Authors:** Christophe Combescure, James A Smith, Christophe Barea, Lotje A Hoogervorst, Rob Nelissen, Perla J Marang-van de Mheen, Anne Lübbeke, the Arthroplasty registry group

**Affiliations:** ^1^ Division of Clinical Epidemiology, Geneva University Hospitals, Geneva, Switzerland; ^2^ Nuffield Department of Orthopaedics, Rheumatology and Musculoskeletal Sciences, University of Oxford, Oxford, United Kingdom; ^3^ Division of Orthopaedic Surgery, Geneva University Hospitals, Geneva, Switzerland; ^4^ Department of Orthopaedics, Leiden University Medical Center, Leiden, Netherlands; ^5^ Safety & Security Science and Centre for Safety in Healthcare, Delft University of Technology, Delft, Netherlands

**Keywords:** total hip arthroplasty, registry, revision, implant, systematic review, meta-analysis

## Abstract

**Purpose:**

- The objective was to investigate the consistency in cumulative revision rates (CRRs) for a selection of total hip arthroplasty cups and stems across national/regional hip arthroplasty registries worldwide.

**Methods:**

- Ten cups and ten stems from total hip systems were randomly selected. Two frequently used implants across registries were added, totalling 11 cups and 11 stems. CRRs and 95% CIs were extracted from the latest annual registry reports using these implants. CRRs were pooled for each cup or stem, and differences between cup-stem combinations and between registries were investigated.

**Results:**

- CRRs were available for ten cups and eight stems from eight registries, totalling 552,148 cups and 727,447 stems. Follow-up was 1–20 years. The 5-year CRR pooled for all cups was 2.9% (95% CI: 2.3–3.6) and for all stems, 3.0% (95% CI: 2.4–3.8). Homogeneous (consistent) CRRs with respect to both associated implant and country were observed for two cups and three stems. Significant differences in CRR were identified in one cup by associated implant only, in one cup by registry only, and in two cups and four stems for both. Sparse data prevented evaluation of four cups and one stem.

**Conclusion:**

- Registries’ annual reports provide a large amount of publicly available information on CRRs of specific implants. These CRRs can be synthesised to improve the assessment of implant performance over time. Our CRR analysis represents a promising approach to detect implants with a consistent low- or high-risk pattern across registries.

## Introduction

Replacement of at least one component of a total hip arthroplasty (THA) – also called revision – is regarded as an important performance indicator of hip arthroplasty. Variation in revision risks may provide important information for clinicians to guide implant selection. Information on the risk of revision over time for a given cup, stem or cup/stem combination are potentially available from the peer-reviewed the medical literature, acknowledged by regulators as a source of clinical evidence regarding medical devices (1), and from annual reports published by national/regional hip arthroplasty registries. The peer-reviewed literature may include early evidence on the performance and safety of implants that were part of obtaining the Conformité Européenne (CE) marking or shortly after market entry. Registries, on the other hand, are monitoring the long-term, real-world performance of implants used in a country/region (2). The new European medical device regulation reinforces the importance of post-market surveillance data and the role of registries (2).

Registries often have procedures to benchmark revision rates at multiple time points to rate the safety and quality of individual implants or categories of implants (e.g. by type of fixation) (3, 4, 5). Because of security and data protection reasons, arthroplasty registries are reluctant or unable to share individual data to be pooled (6) and benchmarking and outlier detection are currently conducted at the national level. The lack of data sharing is a limitation for the detection of outliers and the assessment of an implant’s risk profile. Alternatively, meta-analyses based on aggregate data can be performed to combine registry data, but these have so far mostly been restricted to comparing categories of implants (7, 8, 9).

Combining revision rates of specific implant brands is highly desirable. It would allow testing the consistency of revision results by examining them in different populations and settings improve the precision of the estimated revision rate and increase the potential for stratified analyses. Finally, it would enable pooling of small numbers of implants from different registries and thus facilitate earlier detection of unsafe implants (3, 10). Analyses of combined revision rates for implants would be useful for many stakeholders, including clinicians, hospitals, regulators, notified bodies, manufacturers and health technology assessment agencies. Currently, publications in this area are limited. Hughes *et al.* published specific hip implant revision risks as reported by national and regional arthroplasty registries (11). However, the authors listed revision risks by implant and registry and did not present pooled results by implant.

The objective of this study is to systematically investigate the extent to which the cumulative risk of revision (CRR), for a random selection of currently used total hip stems and cups and for a frequently used cup and stem, is consistent across registries worldwide or varies due to cup-stem combination (associated implant) and geographical location (registry country/region).

## Methods

The systematic review is reported according to the relevant items of the Preferred Reporting Items for Systematic Reviews and Meta-Analyses (PRISMA) statement (12) and it was registered on the Open Science framework (https://osf.io/6gmyx).

### Selection of implants

The selection of the assessed implants is described elsewhere (13, 14). Briefly, ten cups and ten stems were randomly selected from the Orthopaedic Data Evaluation Panel (ODEP) (15) and registry reports (combined list). Six cups were uncemented (Ana.Nova, aneXys (Mathys, Switzerland), EcoFit, Exceed ABT, RM Pressfit Vitamys and Versafit Trio CC) and four were cemented (Cenator, IP X-LINKed, Plasmacup SC and Polarcup (Smith & Nephew, UK)). Nine stems were uncemented (Accolade II, Alloclassic, Avenir (Zimmer Biomet, USA), BiContact, Collomis, Filler, MiniHip, Quadra H and Stelia stem) and one cemented (C-stem AMT). Reported revision risks for these implants were searched in the registry reports. Two implants (Trident cup and Corail stem, both uncemented), which are frequently used internationally in current clinical practice in multiple implant combinations, were added to the search in registries to be able to investigate the CRRs by implant combination.

### Selection of registries and data collection

Registries were eligible if they provided in their annual report the cumulative risk of all-cause revision or the all-cause revision-free survival with 95% confidence intervals (CIs) at any time point for at least one of the 11 stems and 11 cups selected. Using the member list of the International Society of Arthroplasty Registries (ISAR) website (16) and a previous mapping of national and regional registries, the arthroplasty registries reporting annually the CRRs and their 95% CIs by implant were identified (17). Registry country or region and the year of the latest annual report publication were collected. From registry reports published in English language, information was extracted for each cup-stem combination regarding the number of primary THAs recorded (without restriction on diagnosis, age and sex) and the number of revisions for any cause, the corresponding CRRs and the 95% CIs at all reported time points. The initial data search was conducted in July 2021 and an update based on the latest reports was made in November 2023.

### Statistical methods

CRRs were combined across registries by implant at follow-ups of 1, 3, 5, 10 and 15 years using meta-analytic models with random effects and with the restricted maximum likelihood method. The random effect for the cups (respectively stems) associated with the assessed stem (respectively cup) was nested within the random effect for the registry taking into account that the performance of specific cup-stem combinations might vary between registries. If the cup or the stem was reported by a single registry or with a single associated implant, the corresponding random effect was dropped from the model. For the meta-analyses, a complementary log–log transformation was applied to revision-free survivals, and standard errors were derived from the transformed 95% CIs. Pooled estimates were back-transformed and are presented as CRRs. The presence of heterogeneity was investigated with Cochran’s Q test. For a given cup or stem, the relationship between the associated implants and the revision risk was investigated with a multiple meta-regression model with random effects and adjusted for country. With a complementary log–log link function, the exponential of the regression coefficient is an estimate of the hazard ratio (HR) (18). Residual heterogeneity was assessed (Cochran’s Q test and statistic I^2^). Statistical analyses were carried out with software R v4.0.2 (R Core Team (2022). R: A language and environment for statistical computing. R Foundation for Statistical Computing, Vienna, Austria. URL (https://www.R-project.org/) and the package metafor v3.8-1 (19). All statistical tests were two-sided, with a significance level of 0.05.

## Results

Overall, eight arthroplasty registries (six national and two regional) were included. Their list is presented in Appendix 1 (see section on Supplementary materials given at the end of the article) and the flowchart of their selection in Appendix 2. Most of them are in Europe. Six additional registries reporting revision information by implant were identified but were not included because of a different definition of the outcome, a specific rather than the total population, incomplete reporting of CRR (only graphically reported or lack of CIs) or reporting HRs without the underlying rates. Information in at least one of the eight registries was found for 10 of the 11 selected cups (90.9%) and 8 of the stems (72.7%). Seven stems and five cups were assessed by more than one registry. For the randomly selected implants, the sample sizes were larger than 10,000 for six stems and three cups (Table 1). The largest sample sizes were for the Versafit Trio CC cup (48,313 implants, four registries) and the C-stem AMT (70,823 implants, four registries). The length of follow-up varied considerably across implants (from 3 to 19 years for cups and from 7 to 15 years for stems), as well as the number of associated implants (from 1 to 5 stems associated with a given cup and from 3 to 7 cups associated with a given stem). For the frequently used Trident cup and the Corail stem, which were added to the list of randomly selected implants, information was available for a much larger group of patients (391,475 and 427,313 prostheses) in six and eight registries, respectively, with a long follow-up and large numbers of associated implants.

**Table 1:** Implants reported by the registries.

	Registries (*n*)	Primary THAs (*n*)	Associated implants (*n*)	Longest FU included (years)
Cup				
Ana.Nova	1	7,616	2	5
AneXys	1	3,072	2	3
Cenator	1	2,528	1	15
EcoFit	1	2,693	2	5
Exceed ABT	4	54,633	4	15
IP X-LINKed	2	2,099	1	5
Plasmacup SC	1	5,996	2	5
Polarcup	0			
RM Pressfit Vitamys	3	33,723	4	10
Versafit Trio CC	4	48,313	6	10
Trident*	7	391,475	13	15
Stem				
Accolade II	7	96,944	5	10
Alloclassic	3	36,835	7	15
Avenir	6	41,543	5	10
BiContact	1	16,454	4	5
Collomis	0			
Corail*	8	427,313	14	15
C-stem AMT	4	70,823	6	15
Filler	0			
MiniHip	3	5,863	3	10
Quadra H	3	31,672	3	10
Stelia stem	0			

* Selected as frequently used.

THA, total hip arthroplasty; FU, follow-up.

### Risk profile of the selected implants

**Figure 1:**
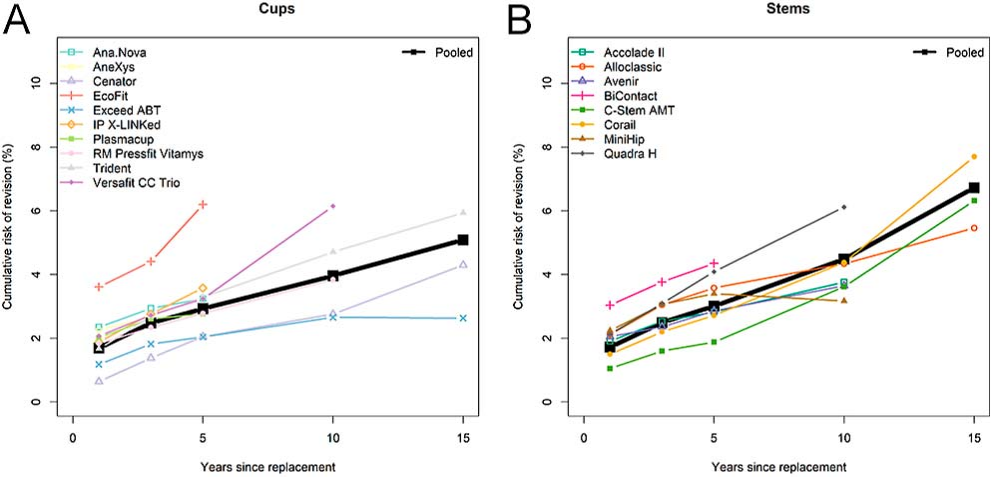
Overall pooled CRRs for cups (A; left panel) and for stems (B; right panel). The black squares represent the overall pooled CRRs and the colored symbols represent the pooled CRRs across registries for specific implants.

The CRR pooled across all selected cups was 1.7% at one year of follow-up and increased to 2.5% at 3 years, 2.9% at 5 years, 4.0% at 10 years and 5.1% at 15 years (Fig. 1A, Table 2). Various implant-specific patterns over time were observed: for some cups, the 1-year CRR was low (e.g., Exceed ABT and RM Pressfit Vitamys) or the increase over time was low (e.g. IP X-LINKed and Plasmacup SC) (Fig. 1A). Pooling the CRRs across registries confirmed the variability between implants (Table 2). At 3 years, the CRR was highest for the EcoFit cup (and the lowest for the Cenator. For implants with long-term data available, differences became more apparent after 10 years, reflecting that the increase of CRR over time was variable. Of the three cups with a long follow-up, the 15-year pooled CRRs varied between 2.6% (Exceed ABT) and 5.9% (Trident; Stryker, USA); the EcoFit cup with the highest 5-year revision risk, had no longer-time data available. The differences in CRRs between cups were detected by meta-regression at 5 (*P* = 0.025) and 10 years (*P* = 0.009).

**Table 2:** CRR at 1, 3, 5, 10 and 15 years combined across registries by implant. Data are presented as pooled CRR (95% CI).

Implant	1 year	3 years	5 years	10 years	15 years
All cups	1.69 (1.25–2.28)	2.48 (1.92–3.21)	2.93 (2.35–3.65)	3.96 (2.86–5.49)	5.09 (3.00–8.55)
Ana.Nova	2.35 (1.26–4.37)	2.94 (1.58–5.44)	3.24 (1.61–6.50)		
AneXys	2.26 (0.89–5.67)	2.66 (1.24–5.64)			
Cenator	0.64 (0.39–1.04)	1.38 (0.99–1.93)	2.05 (1.55–2.70)	2.76 (2.15–3.53)	4.30 (3.38–5.46)
EcoFit	3.61 (2.10–6.17)	4.41 (2.29–8.40)	6.20 (3.94–9.69)		
Exceed ABT	1.18 (0.93–1.50)	1.82 (1.42–2.32)	2.04 (1.67–2.49)	2.66 (1.97–3.57)	2.63 (2.36–2.93)
IP X-LINKed	1.88 (1.07–3.32)	2.76 (1.94–3.93)	3.58 (2.66–4.82)		
Plasmacup	2.02 (1.52–2.67)	2.61 (2.22–3.08)	2.76 (2.37–3.21)		
Polarcup					
RM Pressfit Vitamys	1.83 (1.58–2.11)	2.34 (1.96–2.78)	2.78 (2.31–3.34)	3.84 (2.81–5.25)	
Trident	1.67 (1.11–2.51)	2.69 (1.90–3.80)	3.29 (2.43–4.46)	4.71 (3.07–7.19)	5.94 (3.70–9.47)
Versafit CC Trio	2.07 (1.57–2.72)	2.73 (2.15–3.48)	3.24 (2.56–4.10)	6.15 (5.14–7.36)	
All stems	1.72 (1.19–2.50)	2.49 (1.88–3.31)	3.01 (2.40–3.77)	4.49 (3.32–6.05)	6.72 (3.23–13.72)
Accolade II	1.91 (1.28–2.85)	2.53 (1.84–3.47)	2.83 (2.00–3.98)	3.76 (1.20–11.49)	
Alloclassic	2.12 (1.20–3.72)	3.05 (2.07–4.47)	3.58 (2.61–4.89)	4.34 (3.43–5.48)	5.46 (3.45–8.59)
Avenir	2.05 (1.21–3.47)	2.36 (1.43–3.89)	2.86 (1.91–4.26)	3.65 (2.65–5.03)	
BiContact	3.04 (2.30–4.01)	3.77 (2.83–5.02)	4.35 (2.89–6.54)		
C-stem AMT	1.05 (0.61–1.80)	1.60 (1.02–2.51)	1.88 (1.16–3.05)	3.62 (1.66–7.78)	6.32 (1.80–20.90)
Collomis					
Corail	1.50 (1.06–2.13)	2.20 (1.66–2.90)	2.72 (2.10–3.51)	4.38 (3.04–6.30)	7.70 (4.60–12.73)
Filler					
MiniHip	2.24 (1.35–3.72)	3.06 (1.92–4.87)	3.40 (2.18–5.30)	3.17 (2.60–3.85)	
Quadra H	2.13 (1.75–2.60)	3.09 (2.63–3.63)	4.09 (3.09–5.40)	6.12 (4.97–7.53)	
Stelia stem					

CRR, cumulative revision rates; CI; confidence interval.

For stems, the CRR pooled across all selected implants was 1.7% at 1 year of follow-up and increased to 2.5% at 3 years, 3.0% at 5 years, 4.5% at 10 years and 6.7% at 15 years (Fig. 1B, Table 2). At 1 year, CRRs differed significantly (*P* = 0.004) between implants at 1 year, e.g., three times higher for Bicontact than for C-stem (Fig. 1B, Table 2). The increase over time also varied between implants. It either increased more strongly from the start, e.g. Quadra H or after 5–10 years, e.g. C-stem and Corail. Of the three stems with long follow-up, the 15-year pooled CRRs ranged from 5.5% (Alloclassic) to 7.7% (Corail). In addition to the 1-year difference, meta-regression detected further differences at 3 (*P* = 0.046) and 15 years (*P* = 0.011).

### Differences/consistency by cup-stem combination and/or registries for the selected implants

**Figure 2:**
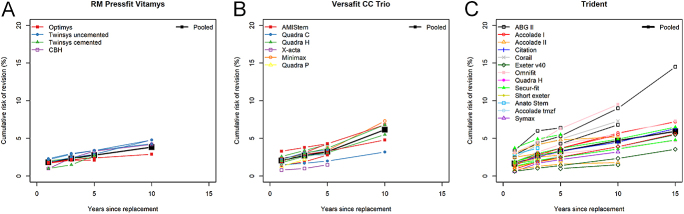
Pooled CRRs (black squares) and CRRs published in annual registries reports for the cups - (A) RM Pressfit Vitamys, (B) Versafit CC Trio and (C) Trident depending on stem combination (colored symbols).

Homogeneous CRRs were observed for the cups IP X-LINKed and Plasmacup, and a single CRR over time was reported for Cenator. For three cups (Ana.Nova, aneXys and EcoFit), the source of the detected heterogeneity could not be investigated due to the small number of CRRs published. For the other cups, three differed significantly by associated implant (RM Pressfit Vitamys, Trident and Versafit CC Trio) and three by registry (Exceed ABT, Trident and Versafit CC Trio) (Appendix 3). For instance, CRRs for the RM Pressfit Vitamys were higher at 5 and 10 years when associated with Twinsys uncemented (pooled 10-year CRR: 4.7%, 95% CI: 4.1–5.4) than with Optimys (10-year CRR: 2.9%, 95% CI: 2.2–3.6) (Fig. 2A), and the associated stems fully explained the apparent heterogeneity and the CRRs were homogeneous for each implant combination between the different registries. In contrast, for Versafit Trio CC, the effect of the associated stems on the CRR was detected only at 5 years (Fig. 2B, Appendix 3). At 10 years, although the results differed visually in particular for the Quadra C – only one registry reported it and with a large CI – no difference was detected. Regarding the Trident, CRR was highest with the associated stems ABGII (pooled 10-year CRR: 8.2%, 95% CI: 6.4–10.6) and Omnifit (pooled 10-year CRR: 6.5%, 95% CI: 3.1–13.5) (Fig. 2C) and lowest with Exeter v40 (pooled 10-year CRR: 2.8%, 95% CI: 1.7–4.4). The CRRs over time by associated stem are displayed in Appendix 4 for the other cups.

The meta-regression model for Trident for the 5-year CRR confirmed that the CRR adjusted for the registry country was higher for the associated stems ABGII (HR = 2.4), Corail (HR = 2.2) and Omnifit (HR = 1.8) than with Exeter v40, and that the CRR adjusted for the associated stem was higher in Finland (HR = 2.9), the Netherlands (HR = 2.0) and Germany (HR = 1.9) than in the United Kingdom (UK) (Table 3). The residual heterogeneity, that is the heterogeneity unexplained by registry or associated implant, remained high for the Trident cup (Appendix 3).

**Table 3:** Multivariable model for the 5-year CRR with the cup Trident.

	Implants, *n*	Adj HR (95% CI)	*P*-value
Registry country/region			
England, Wales	196,048	1 (ref.)*	0.006**
Australia	141,187	1.51 (1.06–2.15)	0.021
Finland	1,877	2.95 (1.59–5.47)	0.001
Germany	6,034	1.90 (1.12–3.21)	0.017
Emilia Romagna, Italy	1,475	1.13 (0.59–2.17)	0.711
Michigan	27,856	1.59 (1.01–2.51)	0.045
Netherlands	11,179	1.98 (1.21–3.23)	0.006
Associated stem			
Exeter v40	233,284	1 (ref.)*	0.005**
ABG II	3,460	2.43 (1.45–4.07)	0.001
Accolade I	44,196	1.54 (0.99–2.38)	0.054
Accolade II	66,441	1.11 (0.73–1.70)	0.614
Accolade tmzf	913	1.17 (0.54–2.52)	0.696
Citation	1,147	1.48 (0.76–2.88)	0.243
Corail	588	2.22 (1.07–4.59)	0.031
Omnifit	5,768	1.77 (1.10–2.85)	0.019
Quadra H	712	1.48 (0.73–3.03)	0.28
Secur-fit	22,987	1.46 (0.93–2.30)	0.102
Short exeter	4,087	1.12 (0.59–2.11)	0.728
Symax	2,073	0.75 (0.35–1.62)	0.464

* Registry country/region and associated stem with the largest sample size selected as the category of reference.

** Statistically significant *P*-values.

Adj, adjusted; CRR, cumulative revision rates.

**Figure 3:**
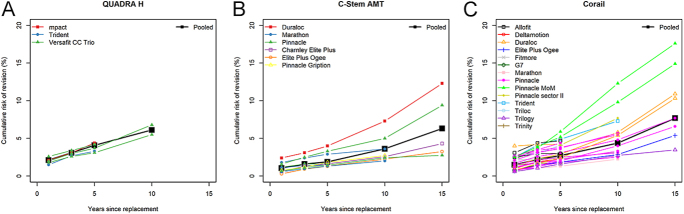
Pooled CRRs (black squares) and CRRs published in annual registries reports for the stems - (A) QUADRA H, (B) C-Stem AMT and (C) Corail depending on cup combination (colored symbols).

Homogeneous CRRs with respect to the associated implant and country were observed for the stem Quadra H (Fig. 3A), Minihip and Avenir. For one stem (Bicontact) the source of the detected heterogeneity could not be investigated due to the small number of CRRs published. All other stems differed significantly by associated cup. For four stems (Accolade II, Alloclassic, C-stem AMT and Corail) the results differed significantly by registry (Appendix 3).

The patterns of CRRs for specific stems over time could vary depending on the associated cup. For instance, the CRRs were higher for the C-stem AMT when associated with the Duraloc (15-year CRR: 12.3% 95% CI: 10.0–15.1) The associated cups Marathon, Charnley Elite plus and Elite plus ogee presented similar patterns with lower CRRs (Fig. 3B). For the frequently used Corail stem, the CRR at long follow-up reached a high level when associated with Duraloc (pooled 15-year CRR: 10.4%, 95% CI: 9.5–11.5) and with Pinnacle MoM (pooled 15-year CRR: 16.6%, 95% CI: 14.3–19.4) (Fig. 3C). Similar trajectories of these two implants up to 15 years were found in two different registries. The CRR differences between the associated cups became more visible after 10 years. CRRs over time by associated cup and by registry location are displayed in Appendix 5 for the other stems.

The meta-regression model on the 5-year CRR adjusted for the registry country/region showed that the CRR was lower with the Marathon (HR = 0.7) and Trinity (HR = 0.5) than with the Pinnacle (Table 4). It also showed that the CRR was higher in Australia (HR = 1.5), Finland (HR = 1.7) Germany (HR = 2.0) and Switzerland (HR = 1.6) than in the UK. The residual heterogeneity, i.e. the heterogeneity unexplained by registry country or associated implant, remained high for the Corail stem (Appendix 3).

**Table 4:** Multivariable model for the 5-year CRR with the stem Corail.

	Implants, *n*	Adj HR (95% CI)	*P*-value
Registry country/region			
England, Wales	241,750	1 (ref.)*	<0.001**
Australia	68,443	1.55 (1.16–2.08)	0.003
Finland	2,184	1.68 (1.12–2.53)	0.012
Germany	42,565	1.98 (1.50–2.62)	<0.001
Emilia Romagna, Italy	1,065	1.83 (0.61–5.51)	0.283
Michigan	3,148	0.99 (0.64–1.54)	0.975
Netherlands	43,606	1.16 (0.85–1.58)	0.357
Switzerland	23,931	1.56 (1.15–2.10)	0.004
Associated cup			
Pinnacle	385,081	1 (ref.)*	0.005**
Allofit	1,987	0.91 (0.61–1.35)	0.635
Deltamotion	1,353	0.75 (0.44–1.28)	0.291
Duraloc	6,008	1.10 (0.83–1.45)	0.499
elite plus ogee	3,188	0.93 (0.60–1.44)	0.752
Fitmore	514	0.75 (0.37–1.50)	0.414
Marathon	19,719	0.66 (0.45–0.96)	0.029
Pinnacle MoM	2,148	1.76 (1.22–2.55)	0.002
Pinnacle Sector II	719	1.14 (0.35–3.75)	0.827
Trident	588	1.62 (0.91–2.88)	0.102
Triloc	842	1.05 (0.60–1.85)	0.854
Trilogy	3,319	0.80 (0.52–1.26)	0.339
Trinity	1,226	0.55 (0.31–0.99)	0.048

* Registry country/region and associated cup with the largest sample size selected as the category of reference.

** Statistically significant *P*-values.

Adj, adjusted; CRR, cumulative revision rates; HR, hazard ratio.

## Discussion

This review shows that graphical representations and quantitative syntheses of reported CRRs, by applying statistical methods for meta-analyses, can be used to facilitate the assessment of implant risk profiles over time from multiple registries. We identified differences in the performance of selected implants and, more importantly, in the performance of cup-stem combinations. The other source of heterogeneity was the registry, which can be stratified by and adjusted for in meta-regression in case of sufficient data. Although the amount of data published by registries is an advantage, it makes the appraisal and synthesis of the risk of revision of the many implants challenging; the graphical representation and the meta-analyses of the CRRs over time we propose here are helpful to identify global patterns. The CRRs varied importantly by implant. For instance, for some cups, the 10-year CRRs were around 3% and around 7% for others. The difference in patterns mainly became more apparent after 5 years.

The overall pooled CRRs at 5 and 10 years in our study (2.9 and 4.0% for cups; 3.0 and 4.5% for stems) were comparable to the all-construct survivorship estimates reported by Paxton *et al.* in 2019 (Sweden 5-year 97.8% and 10-year survival 95.8%, United States 5-year 97.0% and 10-year 95.2%, and Australia 5-year 96.3% and 10-year 93.5%) (20). The pooled all-construct survivorship at 15 years derived from registry data from Australia, Denmark, Finland, New Zealand, Norway and Sweden including patients operated up to 2017 was 89.4% (95% CI 89.2–89.6) (8). Our pooled CRRs of 5.1% for cups and 6.7% for stems at 15 years including THAs operated up to 2022 were lower, which might be related to the inclusion of more recent implants and the fact that differences become more apparent at longer follow-up. An overall decrease in revision rates by year since 2008 has been reported by the National Joint Registry (21) and the Dutch Arthroplasty Register (LROI) (22).

### Limitations

For some of the implants (four cups and one stem) it was not possible to investigate the potential sources of heterogeneity due to the low number of registries reporting CRRs. The inclusion of additional registries would increase the amount of data and allow going further in their assessment. The potential sources of heterogeneity we investigated were limited to the associated implants and the country, but the heterogeneity in CRRs may also be due to inaccurate or insufficiently granular implant labelling leading to grouping of heterogeneous implants under one name, differences in case mix between countries and implant combinations, differences in associated bearing surface, differences in surgical techniques used (especially surgical approach), and health system/policy factors that drive the decision to revise among others (20).

The meta-analytic approach we propose is promising, but methodological improvements are necessary. Conducting meta-analyses independently at each time point multiplies statistical testing and may lead to inconsistent pooled CRRs (e.g., decreasing pooled CRRs over time) because the documented time points vary across registries. To address these issues, a statistical method is needed to assess pooled CRRs over time with a single model.

This review has several limitations related to the reporting of CRRs and to the implant identification/labelling in the annual reports of registries. Since CRRs were not available by age and other risk factors for revision, it was not possible to conduct meta-analyses stratified on those factors and to draw fine-tuned conclusions on the risk profile of implants accounting for the risk profile of patients. In addition, case mix of patients between registries, between implants within registries and between implant combinations is a potential source of confounding. The random effects introduced in the statistical models may imperfectly account for the case mix. The differences in CRRs between implants or implant combinations cannot be interpreted as causal relationships, although consistent patterns in several registries suggest causality. In addition, the role of the bearing surface could not be investigated here since CRRs of implant combinations were not systematically reported by bearing surface. Potential implant misclassification caused by imprecise or insufficient implant identification/labelling in the annual reports is also possible (e.g., Corail details regarding collared or non-collared stem not always reported; different versions of associated cup Pinnacle not systematically detailed). Such misclassifications may produce an excess of heterogeneity, which would be addressed with standardised and more granular implant labelling.

### Strengths

Our study highlights the enormous value of prospective nation- or region-wide data collection with high coverage (94–99% for the established registries) and representativity of the sample, with harmonised baseline and outcome data collection (23) and transparent public reporting of implant performance. Half of the registries reported the outcome of interest as CRRs with 95% CIs, which made it possible to pool the data with meta-analytic methods. The graphical representation of CRRs over time by implant combination and the meta-analyses allow for appraisal of risk patterns and testing of their consistency across different registries. This is helpful to identify not only high-risk implants but also implants or implant combinations showing a consistent low-risk pattern that can be used as standard comparators.

### Perspectives

Despite the limitations and the need for additional methodological developments, the approach we propose was able to identify different patterns in cup-stem combination CRRs, in particular from 5 years on. It is promising for the early detection of outliers. This would be even more efficient if the number of registries reporting CRRs with CIs by implant combination increases. For this, further efforts in harmonised registry reporting are needed. Our approach to synthesising survival outcomes is not limited to orthopaedic implants but can also be applied to assess the risk profile of implants across countries in other medical areas (e.g., cardiology).

Finally, CRR variability between registries, as shown in this study, calls for rethinking the process of international benchmarking. The observed CRRs depend not only on the intrinsic performance of the implants but also on the population, surgery-related factors, on country-/region-specific health care practices and access to care. Thus, rating implants in all registries using the same absolute values/limits seems suboptimal. Within-registry benchmarking, assessing the specific implant’s performance against a comparator group (e.g., all contemporary implants in the registry) and examining in a second step whether there is consistency between registries in the implant’s risk pattern over time and in its comparative performance, might be a way forward worth investigating.

## Conclusion

Registries provide a large amount of publicly available information on specific implant CRRs that can be graphically represented and synthesised to investigate the risk profile of implants depending on the associated implant and country. The approach we proposed is promising to detect implant combinations with a consistently low- or high-risk pattern across registries.

## Supplementary materials



## ICMJE Statement of Interest

C Combescure is an associate editor of *EFORT Open Reviews*. He was not involved in the peer-review or editorial process for this paper, on which he is listed as an author. RN is currently secretary general of the European Federation of National Associations of Orthopaedics and Traumatology (EFORT), and a member of the chair registration board of the Dutch Arthroplasty Register (LROI). AL is the current president-elect of the International Society of Arthroplasty Registries (ISAR). CB is currently a member of the SIRIS Hip & Knee Scientific Advisory Board (Swiss Joint Registry). AS is the contract holder for the analysis of the National Joint Registry of England, Wales and Northern Ireland. AS is an unpaid co-chair of the ISAR Benchmarking working group. CC, CB, JAS, LAH, PJM, AL, BB, ML, KM, OM, RH, LNS declare that there is no conflict of interest that could be perceived as prejudicing the impartiality of the research reported.

## Funding Statement

This study was supported by a Horizon 2020 grant from the European Union (project number 965246).

## Data availability

The data used for this study are publicly available in the annual reports of the registries. The links to the reports are provided in the Appendix of the manuscript.
