# Supplementary material for: Cumulative risk of revision after primary total hip arthroplasty in registries internationally: systematic review and meta-analysis of selected hip stems and cups
Source: EFORT Open Rev. 2025 May 5;10(5):277–85. doi: 10.1530/EOR-2024-0020 (PMC12061013; doi:10.1530/EOR-2024-0020)

Appendix 1: Total hip arthroplasty registries included in the review

Name of registry	Type	Established	Link to annual reports available prior to December 8. 2023
Australian Orthopaedic Association National Joint Replacement Registry (AOANJRR)	National	1999	https://aoanjrr.sahmri.com/annual-reports-2023
Dutch Arthroplasty Register (LROI)	National	2007	https://www.lroi-report.nl/ Report 2023
Endoprothesenregister Deutschland (EPRD)	National	2012	https://www.eprd.de/en/downloads/reports (EPRD Annual Report 2022)
Finnish Arthroplasty Register (FAR)	National	1980	https://www2.thl.fi/endo/report/#index (Dynamic site visited on November 2023)
Michigan Arthroplasty Registry Collaborative Quality Initiative (MARCQI)	Regional	2011	https://marcqi.org/dev/wp-content/uploads/2023/01/2022-REPORT-1-23-2023.pdf
National Joint Registry (NJR), United Kingdom	National	2002	https://reports.njrcentre.org.uk/Portals/0/PDFdownloads/NJR%2020th%20Annual%20Report%202023.pdf
Register of Orthopaedic Prosthetic Implants – RIPO Emilia Romagna	Regional	2000	https://ripo.cineca.it/authzssl/Reports.html (Annual report 2018 Regione Emilia – Romagna in English)
Swiss arthroplasty registry (SIRIS)	National	2012	https://www.siris-implant.ch/fr/Downloads&category=16 SIRIS Report 2023

Appendix 2: Flowchart for the selection of total hip arthroplasty registries

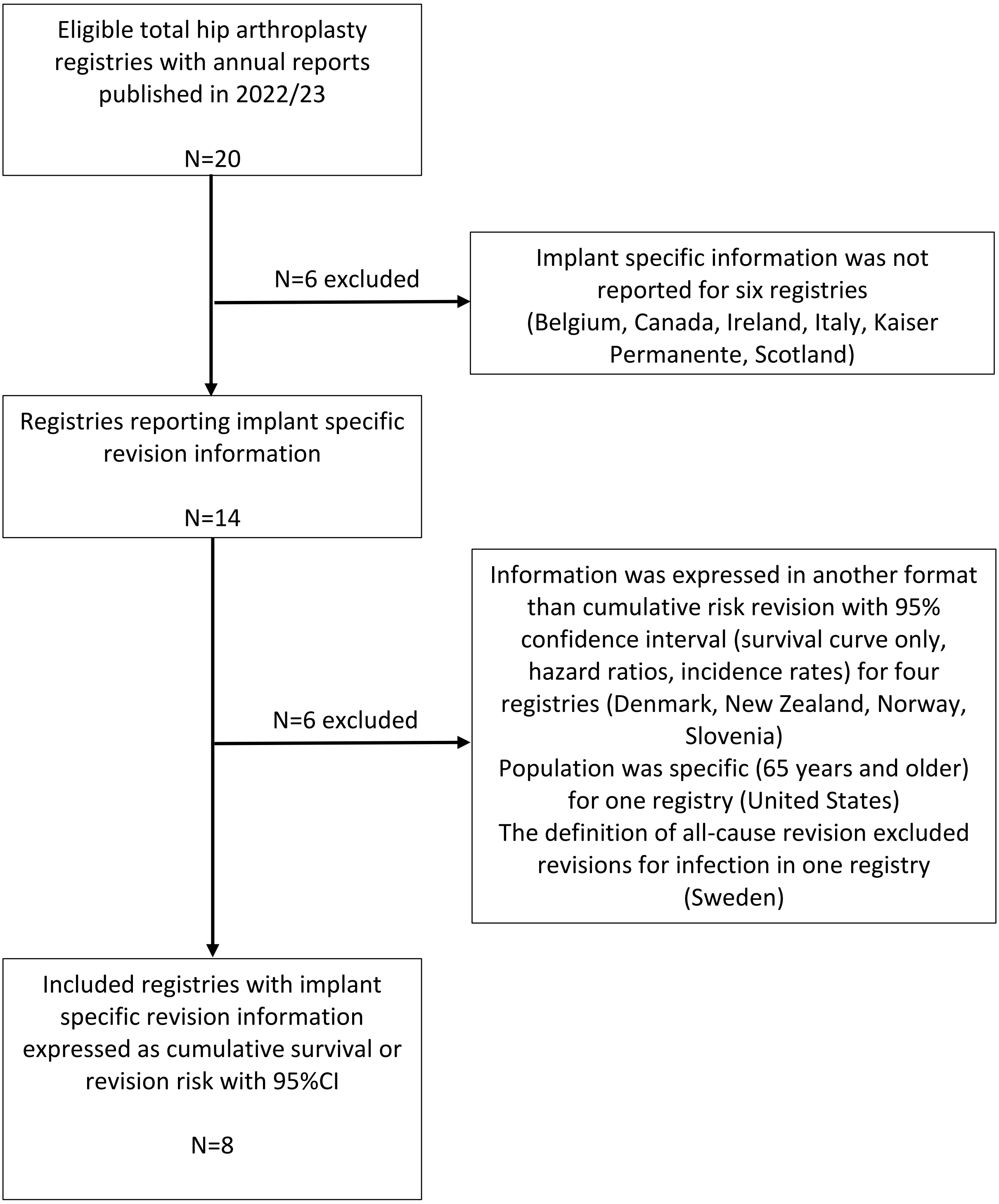

Appendix 3: Heterogeneity, statistical significance of the association between country/associated implant and CRR, and the residual heterogeneity*

	P-value for heterogeneity					P-value for associated implant**					P-value for country registries**					I^2 (%) for residual heterogeneity***				
**Implant**	1y	3y	5y	10y	15y	1y	3y	5y	10y	15y	1y	3y	5y	10y	15y	1y	3y	5y	10y	15y
Cups																				
Ana.Nova	0.006	0.005	0.001																	
AneXys	<0.001	0.001																		
EcoFit	0.051	0.006																		
Exceed ABT	0.006	<0.001	<0.001	<0.001		0.512	0.958	0.893	0.899		0.161	0.012	0.102	0.031			49.3		77.3	
IP X-LINKed	0.084	0.219	0.268																	
Plasmacup	0.4370	0.756	0.836																	
RM Pressfit Vitamys	0.185	0.109	0.033	0.010		0.070	0.014	0.002	0.002		0.367	0.454	0.393	0.768			0.0	0.0	0.0	
Trident	<0.001	<0.001	<0.001	<0.001	<0.001	0.075	0.082	0.005	0.007	0.001	0.004	0.005	0.006	0.012	0.089	90.1	90.7	90	95.6	93.4
Versafit CC Trio	<0.001	<0.001	<0.001	0.021		0.175	0.117	0.033	0.344		<0.001	<0.001	0.005	0.333		29.2	37.6	30.7		
Stems																				
Accolade II	<0.001	<0.001	<0.001	<0.001		0.007	0.095	0.004	<0.001		<0.001	<0.001	<0.001	<0.001		0.0	0.0	0.0	0.0	
Alloclassic	<0.001	<0.001	<0.001	<0.001	0.0001	0.556	0.001	0.170	0.065		0.014	<0.001	0.040	0.690		81.3	24.8	69.7		
Avenir	<0.001	<0.001	<0.001	0.018		0.315	0.414	0.068	0.514		0.046	0.355	0.051	0.282		88.2				
BiContact	<0.001	<0.001	<0.001																	
C-Stem AMT	<0.001	<0.001	<0.001	<0.001	<0.001	0.010	0.262	0.304	0.031	0.7049	<0.001	<0.001	<0.001	<0.001	<0.001	0.0	0.0	0.0	0.0	56.5
Corail	<0.001	<0.001	<0.001	<0.001	<0.001	0.552	0.216	0.005	0.083	<0.0001	<0.001	<0.001	<0.001	0.7565	0.001	57.3	75.2	75.0	93.1	0.0
MiniHip	0.002	0.002	0.003	0.316		0.532	0.266	0.186			0.109	0.423	0.306							
Quadra H	0.008	0.047	0.002	0.069		0.630	0.896	0.467			0.022	0.326	0.812			18.9				

*: heterogeneity that was not explained by the registry nor the associated implant

**: the associations between associated implant (respectively registry) and CRR were assessed only for implants reported with at least three combinations (respectively three registries)

***: the residual heterogeneity was assessed only if an association with the associated implants or with the registries was detected

Appendix 4: CRRs for cups by registry and associated stem. Black squares represent the pooled CRRs independently of the associated stem and coloured symbols represent the associated stems.

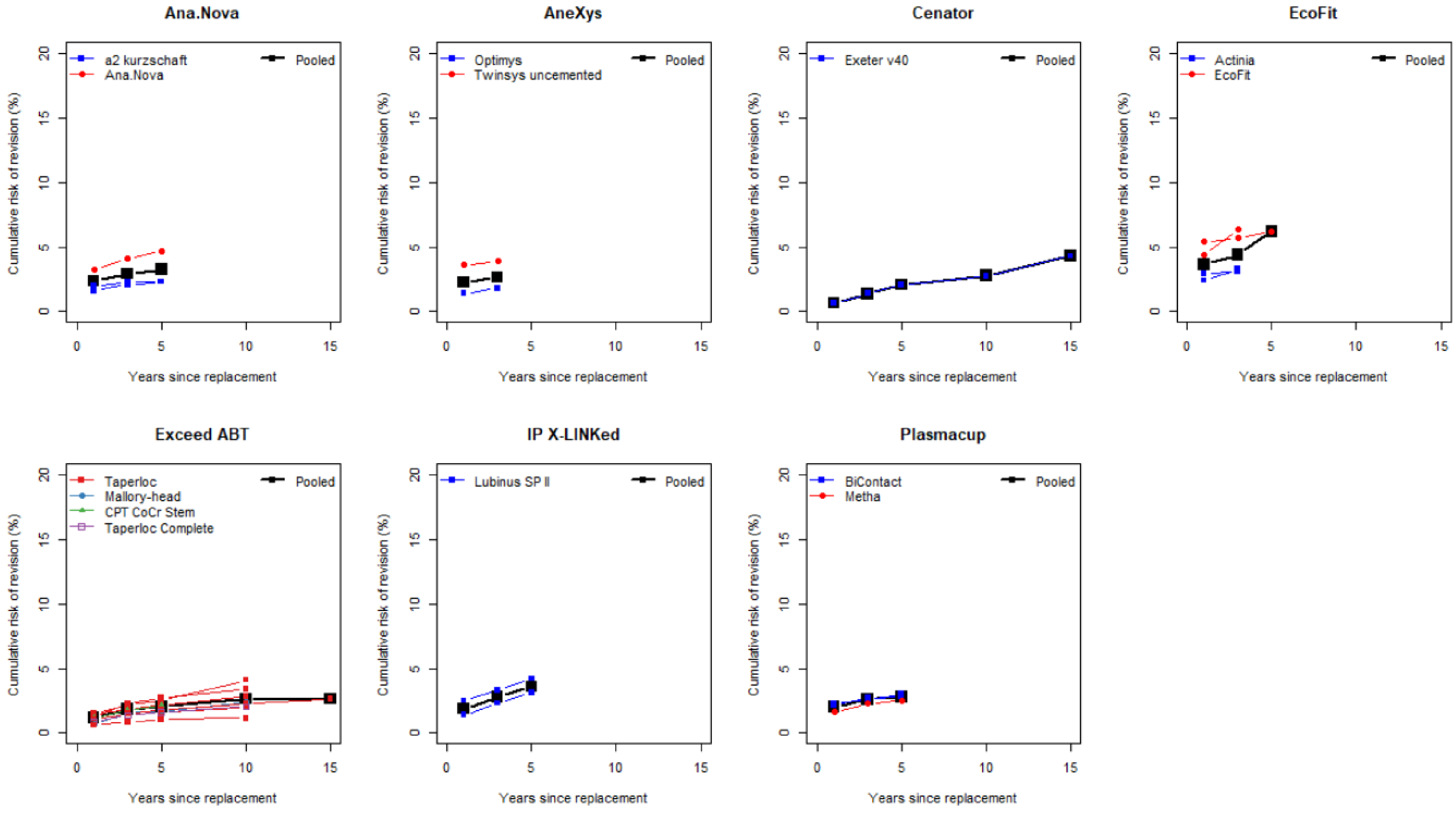

Appendix 5: CRRs for stems by registry and associated cup. Black squares represent the pooled CRRs independently of the associated cups and coloured symbols represent the associated cups.

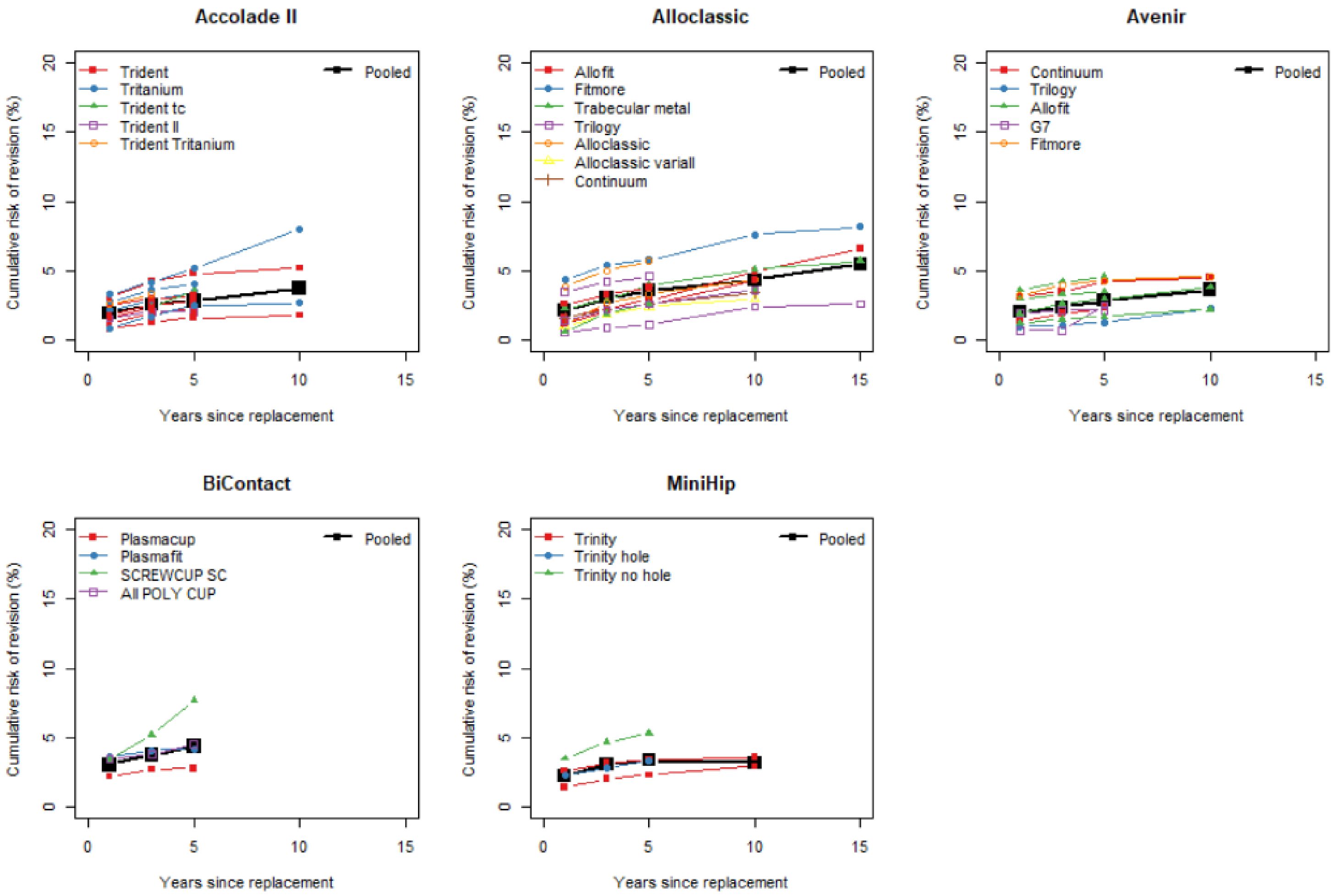

Supplement: Supplementary file 1 [file supplementary_materials.pdf]
